# Dichloro(2,2′-bipyridine)copper/MAO: An Active and Stereospecific Catalyst for 1,3-Diene Polymerization

**DOI:** 10.3390/molecules28010374

**Published:** 2023-01-02

**Authors:** Giovanni Ricci, Giuseppe Leone, Giorgia Zanchin, Francesco Masi, Massimo Guelfi, Guido Pampaloni

**Affiliations:** 1 CNR—Istituto di Scienze e Tecnologie Chimiche “Giulio Natta” (SCITEC), Via A. Corti 12, I-20133 Milano, Italy; 2 Scientific Advisor, Via Galvani 7, I-26866 Sant’Angelo Lodigiano, Italy; 3 Dipartimento di Chimica e Chimica Industriale, Università di Pisa, Via Moruzzi 13, I-56124 Pisa, Italy

**Keywords:** copper, 1,3-diene polymerization, catalysis

## Abstract

Dichloro(2,2′-bipyridine)copper was synthesized by reacting copper dichloride with bypyridine, and its behavior, in combination with methylaluminoxane (MAO), in the polymerization of butadiene, isoprene, 2,3-dimethyl-1,3 butadiene, and 3-methyl-1,3-pentadiene was examined. The purpose of this study is to find catalytic systems that are more sustainable than those currently used for the polymerization of butadiene and isoprene (e.g., Co and Ni), but that are comparable in terms of catalytic activity and selectivity. Predominantly, syndiotactic 1,2 polybutadiene, crystalline syndiotactic 3,4 polyisoprene, crystalline syndiotactic 1,2 poly(3-methyl-1,3-pentadiene), and crystalline *cis*-1,4 poly(2,3-dimethyl-1,3-butadiene) were obtained in a manner similar to that observed with the analogous iron complex. As far as we know, the investigated catalytic system represents the first example of a copper-based catalyst in the field of stereospecific polymerization. Given the great availability of copper, its extremely low toxicity (and therefore high sustainability), and the similarity of its behavior to that of iron, the result obtained seems to us of considerable interest and worthy of further investigation.

## 1. Introduction

**Figure 1:**
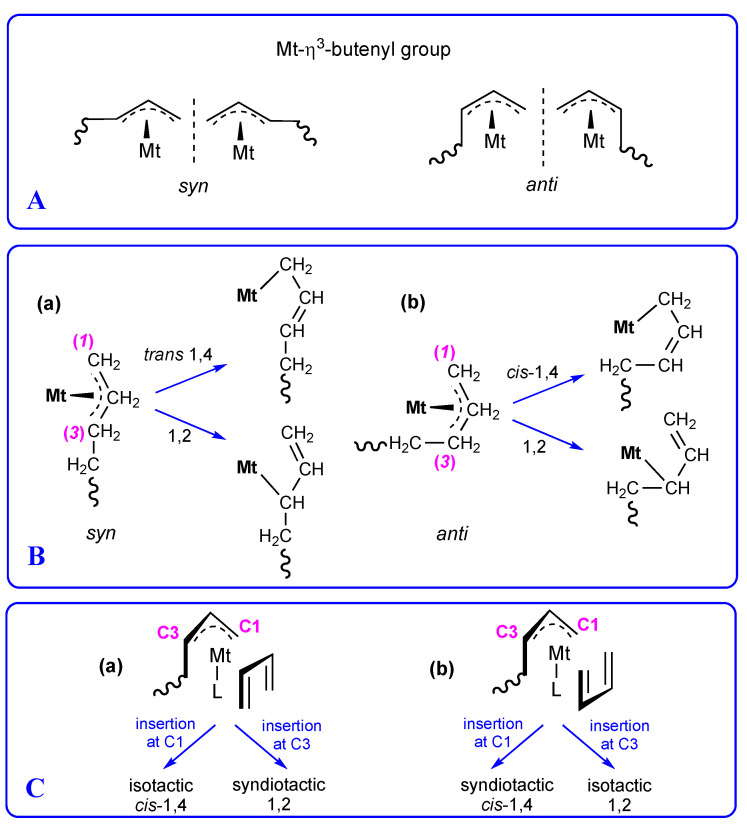
(**A**) Bonds between the growing chain and the transition metal of the catalyst; (**B**) formation of 1,4 vs. 1,2 monomeric units from (**a**) *syn* and (**b**) *anti* Mt-butenyl groups; (**C**) possible orientations ((**a**) *exo-exo* and (**b**) *exo-endo*) of the new incoming monomer with respect to the last-inserted unit (L is a generic ligand) and formation of *cis*-1,4 and 1,2 polymers having a syndiotactic or an isotactic structure.

In recent years, research in the field of the stereospecific polymerization of conjugated dienes [1,2,3] has mainly been directed towards the use as precatalysts of transitional metal and lanthanide organometallic complexes having a well-defined structure [4,5,6,7,8,9,10], with different types of organic ligands containing nitrogen and/or or oxygen and/or phosphorus as donor atoms (e.g., mono and bidentate phosphines, pyridyl-imines, bis-imines, keto-imines). The reason for this interest lies in the fact that the characteristics of the ligands, combined with the nature of the metal to which they are coordinated, are capable of exerting a considerable influence on the polymerization regio- and stereo-selectivity (Figure 1) [11], the molecular weight (M_w_), and the molecular weight distribution (M_w_/M_n_), and, in some cases, they may also impart living features to the catalyst itself [12,13,14,15]. However, the regio- and stereo-selectivity in the polymerization of 1,3-dienes, besides being greatly influenced by the catalytic structure, also strongly depend on the conjugated diene structure (i.e., the presence of substituents on the monomeric unit) [1,11], and ultimately we can state that it is possible to obtain more or less stereoregular polymers from conjugated dienes depending on the right combination/choice of the type of metal, type of ligand, and type of monomer. It is in fact well known that: (1) the same catalytic system can behave differently toward different 1,3-dienes (see for instance the system FeCl_2_(bipy)_2_/MAO (bipy = 2,2′-bypiridine), which gives highly syndiotactic 1,2 polymers from 3-methyl-1,3-pentadiene and highly *cis*-1,4 polymers from 2,3-dimethyl-1,3-butadiene) [16,17]; (2) the same 1,3-diene can give a different polymer depending on the type of catalyst used for its polymerization (see for instance 3-methyl-1,3-pentadiene, which gives a syndiotactic 1,2 polymer when polymerized with FeCl_2_(bipy)_2_/MAO [16,17] and an isotactic 1,2 polymer if polymerized with CoCl_2_(P^n^PrPh_2_)_2_/MAO [18], and 1,3-butadiene, which gives a syndiotactic 1,2 polymer with the system CrCl_2_(dmpe)_2_/MAO (dmpe = 1,2-bis(dimethylphosphino)ethane) and an isotactic 1,2 polymer with CrCl_2_(dmpm)_2_/MAO) (dmpm = bis(dimethylphosphino)methane) [19,20]; (3) the same ligand can give rise to more or less active and selective catalytic systems according to the metal to which it is coordinated (see for instance dmpe; CrCl_2_(dmpe)_2_/MAO is an extremely active and selective catalyst for the polymerization of 1,3- butadiene [19], while FeCl_2_(dmpe)_2_/MAO is practically inactive [4]); and (4) the same metal can provide active and selective catalysts depending on the type of ligand coordinated (see for instance iron; FeCl_2_(dmpe)_2_ is inactive in the polymerization of isoprene [4], FeCl_2_(bipy)_2_/MAO is extremely active in the polymerization of isoprene, giving a highly syndiotactic 3,4-polymer [16,17,21], while pyridyl-imine iron dichloride complexes in combination with MAO are quite active in the polymerization of isoprene, giving poly(isoprene)s with a mixed 1,4/3,4 structure. The 1,4 and 3,4 units are randomly or alternately distributed along the polymer chain depending on the nature of the pyridyl-imine ligand [22,23,24,25,26,27]).

It follows that, in principle, each transition metal or lanthanide could provide an active and selective catalytic system once the right combination of metal, ligand, and diene monomer is chosen.

On the basis of this observation, we have focused our attention on copper. Until now, there had been no reports in the literature on the stereospecific polymerization of 1,3-dienes with copper-based catalysts. Our interest in copper arises from the need to find and test new catalytic systems that have a lower environmental impact (and are consequently more sustainable) and that are capable of replacing, by exhibiting comparable catalytic activities and selectivities, the catalytic systems currently used, based on metals characterized by high toxicity such as cobalt, chromium, and nickel.

To begin, we synthesized the bipyridyl copper dichloride complex and examined its behavior as a precatalyst in the polymerization of various 1,3-dienes. The results obtained turned out to be quite interesting and are shown in the present paper. 

## 2. Results

### 2.1. Synthesis and Characterization of CuCl_2_(bipy)

The reaction of 2,2′-bipyridine with CuCl_2_ or CuCl_2_(H_2_O)_2_ (bipy/Cu molar ratio = 1) to give pure Cu(bipy)Cl_2_ was initially performed in boiling toluene, but we observed that the formation of the complex occurs smoothly at room temperature when using ethanol as a reaction medium [28,29]. The compound is the same either starting from anhydrous or from hydrated copper chloride. 

The obtained Cu(bipy)Cl_2_ compound was a stable turquoise microcrystalline solid and was characterized using analytical and infrared data. The Infrared spectrum is characterized by strong absorptions in the 1600−1500 cm^−1^ range due to the C=N stretching vibrations (Figure S1–S3 in the Supplementary Materials section), which is shifted about 20 cm^−1^ towards the lower wavenumbers with respect to the uncoordinated species. The copper compound is soluble in polar solvents, such as acetonitrile or dichloromethane, and is substantially insoluble in hydrocarbons. 

### 2.2. Polymerization of 1,3-Dienes

The results obtained in the polymerization of 1,3-dienes with the CuCl_2_(bipy)/MAO catalytic system are summarized in Table 1 and can be summarized as follows. 

**Figure 2:**
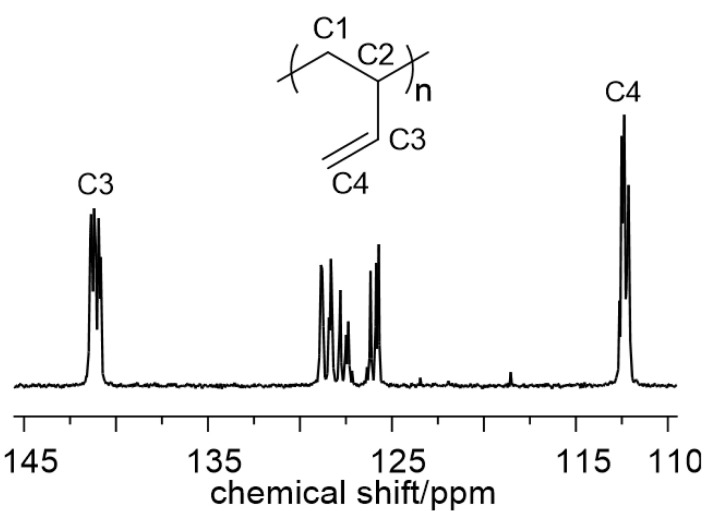
The ^13^C NMR spectrum (olefinic region) of 1,2 syndiotactic poly(1,3-butadiene) obtained with Cu(bipy)Cl_2_/MAO (Table 1, entry 2).

The polymerization of 1,3-butadiene gives polymers with a predominantly 1,2 structures (1,2 content about 65%), a rather low syndiotacticity (percentage of syndiotactic pentads (rrrr) around 30% (Figure 2)), and consequently very low melting points (around room temperature), very high molecular weights (up to 1,900,000 g×mol^−1^), and narrow molecular weight distributions (particularly at room temperature, M_w_/M_n_ from 1.3 to 2.3). The syndiotacticity and the polymer molecular weight decrease with the increase in polymerization temperature from 22 °C to 60 °C (Table 1, entry 3 vs. 4). The polymerization rate is rather low since several hours are needed to reach appreciable monomer conversion. 

**Figure 3:**
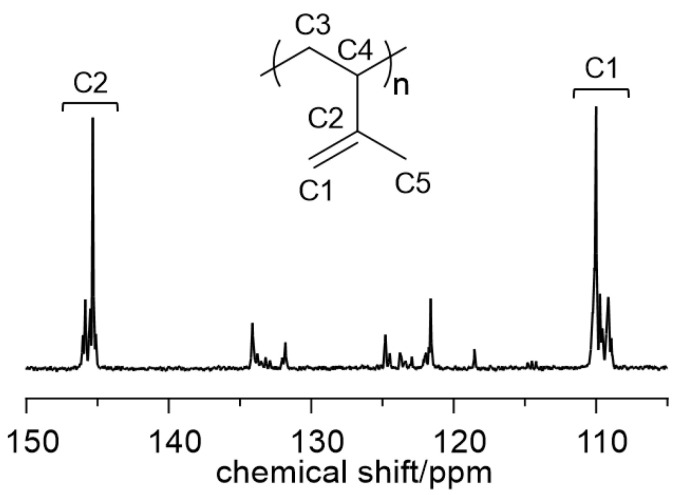
The ^13^C NMR spectrum (olefinic region) of 3,4 syndiotactic poly(isoprene) obtained with CuCl_2_(bipy)/MAO (Table 1, entry 8).

The polymerization of isoprene catalyzed by CuCl_2_(bipy)/MAO, contrary to what has been observed with most catalytic systems based on transition metals and lanthanides, is instead much faster than the polymerization of 1,3-butadiene. Crystalline polymers with an essentially 3,4 syndiotactic structure (Figure 3) (percentage of syndiotactic pentads (rrrr) around 55%) and a melting temperature (T_m_) of up to 115 °C are obtained. The molecular weight of the resultant polymers is very high, and the molecular weight distribution rather narrow (M_w_/M_n_ from 1.4 to 2). The narrow molecular weight distribution, also observed in the case of the polymerization of butadiene, seems to suggest the presence of single-site catalysts.

The Al/Cu molar ratio has some influence on the catalyst activity, which was found to decrease with a decrease in the Al/Cu molar ratio, while a negligible effect was observed on the polymerization selectivity. An increase in the polymerization temperature determines a decrease in the polymerization stereoselectivity as well as a decrease in the polymer molecular weight accompanied by an increase in the molecular weight distribution (Table 1, entry 7 vs. 6).

The CuCl_2_(bipy)/MAO system is also able to polymerize 2,3-dimethyl-1,3-butadiene and *(E)*-3-methyl-1,3-pentadiene. The polymerization of 2,3-dimethyl-1,3 butadiene is quite fast and high conversions are obtained in a few hours. The polymer obtained is insoluble in hot *ortho*-dichlorobenzene and C_2_D_2_Cl_4_, which prevented us from determining its molecular weight and carrying out a structural NMR analysis. However, the melting point (197.5 °C) and the FT-IR spectrum in the solid state of the polymer perfectly correspond to those observed in the case of the *cis*-1,4 poly(2,3-dimethyl-1,3-butadiene) obtained with the catalytic systems CpTiCl_3_/MAO [30], AlEt_2_Cl/Nd(OCOC_7_H_15_)_3_/Al(^i^Bu)_3_ [31], and FeCl_2_(bipy)_2_/MAO [16,17], thus confirming its *cis*-1,4 structure. 

On the other hand, the polymerization of *(E)*-3-methyl-1,3-pentadiene is rather slow. Again, a polymer insoluble in *ortho*-dichlorobenzene and C_2_D_2_Cl_4_ is obtained, preventing GPC and NMR analysis in solution. However, in this case the melting point (241 °C) and the FT-IR spectrum in the solid state also perfectly correspond to those observed in the case of the poly(3-methyl-1, 3-pentadiene) with a syndiotactic 1,2 structure obtained with the catalytic system FeCl_2_(bipy)_2_/MAO [16,17]. The fact that different polymer structures are obtained from butadiene, isoprene, 2,3-dimethyl-1,3 butadiene, and (E)-3-methyl-1,3-pentadiene with the same catalytic system only confirms once more the importance of the monomer structure in the stereoselectivity in the polymerization of conjugated dienes [1,11].

## 3. Discussion

The results obtained in the polymerization of 1,3-butadiene, isoprene, 2,3-dimethyl-1,3-butadiene, and 3-methyl-1,3-pentadiene with the catalyst CuCl_2_(bipy)/MAO are perfectly comparable to those obtained in the polymerization of the same 1,3-dienes with the analogous iron catalyst FeCl_2_(bipy)_2_/MAO [16,17,21], except for the polymerization rate, which was generally much faster in the case of iron.

**Figure 4:**
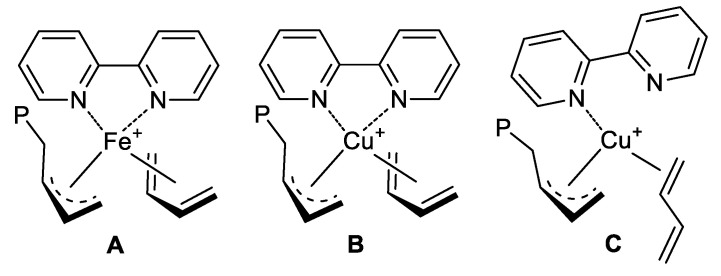
Possible catalytic center structures (**A**–**C**).

The results obtained in the polymerization of 1,3-dienes with the catalyst FeCl_2_(bipy)_2_/MAO were interpreted by admitting the formation of a catalytic center having the structure shown in Figure 4A, i.e., only one bipyridyl ligand coordinated to the iron atom, the monomer coordinated *cis*-η^4^, and the growing chain linked to the iron atom by means of an *anti* η^3^-allyl bond [16,17].

One could imagine a similar structure for the catalytic center in the case of copper (Figure 4B), but, unfortunately, this is not possible as copper would end up with a higher number of electrons (3 excess electrons) than those it can accept (8 electrons) according to the 18 electron rule.

It is indeed necessary to hypothesize a different situation. A plausible one is that shown in Figure 4C: the bipyridyl ligand coordinated to the copper atom by means of only one nitrogen and the monomer *trans*-η^2^ and the growing chain coordinated to the copper atom through a *syn* η^3^-allylic bond as this is the allyl unit that originates from a *trans*-η^2^ coordination of the monomer (an *anti* η^3^-allylic bond originates from a *cis*-η^4^ coordination of the monomer). With such a structure, copper would receive 7 electrons, thereby respecting the 18 electron rule (a 1 electron deficit). However, the structure shown in Figure 3 is not yet able to explain the polymerization data obtained: the polymer obtained from 2,3-dimethyl-1,3-butadiene has a *cis*-1,4 structure, and in the polymers from 1,3-butadiene and isoprene, having respectively a predominant 1,2 and 3,4 structure, the remaining units have only a *cis*-1,4 structure, but while a 1,2 unit and a 3,4 unit can derive from both a *syn* and an *anti* allyl unit, a *cis*-1,4 unit is formed solely from an *anti* allyl unit [1,4,5,11]. It is therefore necessary to hypothesize that the allyl unit of the *syn* type isomerize to an *anti* allyl unit to allow the formation of a *cis*-1,4 unit, and that the occurrence of such isomerization and the frequency with which it occurs may be a function of the type of monomer polymerized.

**Figure 5:**
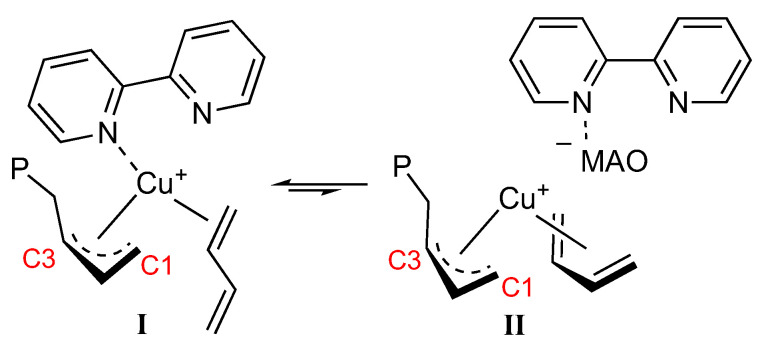
Sketch of the possible equilibrium between form (**I**) and form (**II**).

An alternative explanation is shown in Figure 5. The diene monomer can coordinate with both double bonds (*cis*-η^4^) favoring the displacement of the ligand and its complete migration onto MAO, thus allowing the formation of an *anti* allyl unit which in turn can lead to the formation of a *cis*-1,4 unit through the insertion of the incoming monomer into C1 of the butenyl group. A sort of equilibrium can be hypothesized between the form (I), with the monomer *trans*-η^2^ and the ligand coordinated with only one nitrogen atom, and the form (II), with the monomer *cis*-η^4^ coordinated and the ligand having migrated onto MAO, with the formation of 1,2 (3,4) units through the insertion of the incoming monomer into C3 of the butenyl group rather than *cis*-1,4, depending on whether the equilibrium is more shifted towards form (I) or form (II), respectively.

Obviously, the above interpretations represent only working hypotheses, both plausible in our opinion, but certainly to be further explored through additional computational studies. 

Finally, as mentioned above, the formation of polymers with essentially 1,2 and 3,4 structures from 1,3-butadiene and isoprene, respectively, and the formation of a highly *cis*-1,4 polymer from 2,3-dimethyl-1,3-butadiene and a syndiotatctic 1,2 polymer from 3-methyl-1,3-pentadiene using the same catalytic system once more highlights the fundamental role played by the monomer structure in determining the polymerization selectivity [1,11].

## 4. Materials and Methods 

### 4.1. General Procedures and Materials

Anhydrous copper dichloride (Merck, 99.9% pure), copper chloride dihydrate (Merck, reagent grade), 2,2′-bipyridine (Supelco, ACS reagent), methylaluminoxane (MAO) (Merck, 10 wt% solution in toluene), and deuterated solvent for NMR measurements (C_2_D_2_Cl_4_) (Merck, >99.5% atom D) were used as received. Commercial ethyl alcohol (Merck, 96% pure) was degassed under vacuum, the flask was then filled with dry dinitrogen, and the solvent was stored over molecular sieves. Diethylether (Merck, 99% pure) was refluxed over Na/K alloy for ca. 8 h, distilled, and stored over molecular sieves under dry dinitrogen. Toluene (Merck, 99.8% pure) was refluxed over Na for ca. 8 h, then distilled and stored over molecular sieve under dry dinitrogen. Prior to each run, 1,3-Butadiene (Merck, ≥99%) was evaporated from the container, dried by passing through a column packed with molecular sieves, and condensed into the reactor which had been precooled to –20 °C. Isoprene (Merck, ≥99.5%), 2,3-dimethyl-1,3-butadiene (Fluka, ≥96), and 3-methyl- 1,3-pentadiene (Merck, 99% pure, mixture of (Z) and (E) isomers) were refluxed over calcium hydride for 3 h, distilled trap-to-trap, and stored under dry nitrogen. 

### 4.2. Synthesis of CuCl_2_(bipy)

The compound was prepared according to a slight modification of the procedure outlined in the literature [28]. An ethanol solution (25 mL) of bipy (2.330 g, 0.015 mmol) was added to a solution of copper chloride dihydrate (2.532 g, 0.015 mol) in ethanol (45 mL), and the resulting solution was stirred for 60 min. During the stirring, a large amount of solid precipitated from the solution. The mixture was stirred at room temperature for an additional 5 h. The turquoise-colored precipitate was recovered by filtration, washed with ethanol and diethyl ether, and dried in vacuo at room temperature, affording 4.011 g (93 % yield) of Cu(C_10_H_8_N_2_)Cl_2_ as a turquoise microcrystalline solid. Elemental analysis (%): Calc. for C_10_H_8_Cl_2_CuN_2_: C, 41.32; H, 2.77; N, 9.34; Cl, 24.40; Cu, 21.86. Found: C, 41.64; H, 2.61; N, 9.20; Cl, 24.00; Cu, 21.60. Selected IR data (solid state, cm^−1^): 3109 w, 3053 w, 1602 ms, 1445 s, 1318 mw, 1158 m, 1025 m, 908 mw, 775 vs, 729 vs. 

### 4.3. Polymerization of 1,3-Dienes

Polymerizations were carried out in a 25 mL round-bottomed Schlenk flask. A standard procedure is reported. Prior to starting the polymerization, the reactor was heated to 110 °C under vacuum for 1 h and backfilled with nitrogen. The 1,3-Butadiene was condensed into the Schlenk flask kept at −20 °C, toluene was added, and the solution was brought to the desired polymerization temperature. MAO and a toluene solution (2 mg/mL) of the copper complex were then added in that order. The polymerization was stopped with methanol containing a small amount of hydrochloric acid. The polymer obtained was then coagulated by adding 40 mL of a methanol solution containing 4% Irganox^®^ 1076 antioxidant and HCl, repeatedly washed with fresh methanol, and finally dried under vacuum at room temperature to a constant weight. The polymerizations with isoprene, 2,3-dimethyl-1,3-butadiene, and 3-methyl-1,3-pentadiene were carried out in the same way.

### 4.4. Polymer Characterization

Attenuated total reflectance (ATR)-Fourier transform infrared spectroscopy (FTIR) spectra were recorded at room temperature in the 600–4000 cm^−1^ range with a resolution of 4 cm^−1^ using a Perkin Elmer Spectrum Two spectrometer. NMR spectra were recorded on a Bruker NMR advance 400 Spectrometer operating at 400 MHz (1H) and 100.58 MHz (^13^C) in the PFT mode at 103 °C. NMR samples were prepared by dissolving from 60 mg to 80 mg of polymer in about 3 mL of C_2_D_2_Cl_4_ in 10 mm probes, and hexamethyldisiloxane (HMDS) was referred to as the internal standard. The relaxation delay was 16 s. The molecular weight average (M_w_) and the molecular weight distribution (M_w_/M_n_) were obtained using a high temperature Waters GPCV2000 size exclusion chromatography (SEC) system equipped with a refractometer detector. The experimental conditions consisted of three PL Gel Olexis columns, *ortho*-dichlorobenzene (DCB) as the mobile phase, a flow rate of 0.8 mL/min, and a temperature of 145 °C. The calibration of the SEC system was achieved using eighteen narrow M_w_/M_n_ PS standards with molar weights ranging from 162 g/mol to 5.6 × 10^6^ g/mol. For the SEC analysis, about 12 mg of polymer was dissolved in 5 mL of DCB with 0.05% of BHT as antioxidant. The microstructure of the resultant polymers (i.e., *cis*-1,4 unit content (%) and 1,2 (3,4 in the case of isoprene) unit content (%); syndiotactic index (rrrr%) of the 1,2 poly(1,3-butadiene)s and of the 3,4 poly(isoprene)s) was determined by ^1^H and ^13^C NMR, in accordance with the literature [17,32,33,34,35,36].

## 5. Conclusions

To the best our knowledge, for the first time in the field of the stereospecific polymerization of 1,3-dienes, a copper system, obtained by combining CuCl_2_(bipy) and MAO, was reported to polymerize 1,3-dienes, providing highly crystalline 3,4 syndiotactic poly(isoprene), *cis*-1,4 poly(2,3-dimethyl-1,3-butadiene), syndiotactic 1,2 poly(3-methyl-1,3-pentadiene), and predominantly 1,2 poly(1,3-butadiene), exhibiting a moderate activity and selectivity. In view of these rather encouraging first results, further work is currently underway, and other copper complexes with different organic ligands are being considered in order to evaluate the possibility of obtaining more active and selective catalytic systems, as happened previously in the case of titanium- and zirconium- [37,38], vanadium- [39,40], chromium- [19,20], iron- [22,23,24,25,26,27], cobalt- [41], and neodymium-based catalytic systems [42].

Furthermore, syndiotactic 1,2 poly(1,3-butadiene) and syndiotactic 3,4 poly(isoprene) are polymers of potential industrial interest, and these new copper-based catalysts, in light of the natural abundance of copper, its low toxicity, and low environmental impact, could represent a valid alternative to other catalytic systems, such as those based on cobalt, currently used for their production.

## Figures and Tables

**Table 1:** Polymerization of 1,3-Dienes with CuCl_2_(bipy)/MAO Catalyst ^a^.

M ^b^	Entry	Al/Cu	Time (h)	Yield (%)	*cis*-1,4 ^c^ (%)	1,2 ^c^ (%)	3,4 ^c^ (%)	[rrrr] ^d^ (%)	T_m_^e^ (°C)	T_c_^e^ (°C)	T_g_^e^ (°C)	M_w_^f^ (g/mol)	M_w_/M_n_^f^
B	1	1000	4	10	34.2	65.8		31.3	22	5	−46	1,319,100	1.3
	2	1000	20	42	33.8	66.2		32.6	30	7	−47	1,900,000	1.7
	3 ^g,h^	500	4	35	34.9	65.1		25.8	nd	nd	nd	633,800	4.6
	4 ^g^	500	24	52	35.1	64.9		30.9	24	6	−46	1,442,600	2.3
IP	5	1000	0.5	8	24.1		75.9	49.5	113.8	78	5.4	910,000	1.6
	6	1000	5	57	25.7		74.3	56.9	114.9	76.5	9.4	1,668,750	2.0
	7 ^h^	1000	5	35	31.8		68.2	40.5	45.4		−1.2	457,970	3.7
	8 ^g^	500	4	50	24.4		75.6	57.6	113	77	8.1	2,473,140	1.4
	9 ^i^	100	2	35	25.1		74.9	54.4	103	64	9.6	2,922,670	1.6
DMB	10	1000	4	76	≥99				197.5	170	−4.1	nd	nd
3MP	11	1000	24	19		≥99			241			nd	nd

^a^ polymerization conditions: monomer, 2 mL; toluene, total volume 16 mL; Cu, 10 μmol; temperature, 22 °C; ^b^ M = monomer; B = 1,3-butadiene; IP = isoprene; DMB = 2,3-dimethyl-1,3-butadiene; 3MP = 3-methyl-1,3-pentadiene; ^c^ determined by ^1^H NMR; ^d^ syndiotacticity index (percentage of syndiotactic pentads), determined by ^13^C NMR; ^e^ melting temperature (T_m_), crystallization temperature (T_c_) and glass transition temperature (T_g_), determined by DSC; ^f^ determined by SEC; ^g^ Cu, 20 μmol; ^h^ polymerization temperature, 60 °C; ^i^ Cu, 30 μmol; nd = not determined.

## Data Availability

Not applicable.

## Supplementary Materials

The following supporting information can be downloaded at: https://www.mdpi.com/article/10.3390/molecules28010374/s1, FT-IR spectra of the copper complex Cu(bipy)Cl_2_ (Figure S1–S3); FT-IR spectra (Figure S4–S13); ^1^H and ^13^C NMR spectra (Figure S14–S22) of the polymers of Table 1.

## Sample Availability

Samples of the copper compound and of the polymers are not available from the authors.

## References

[1] Porri L., Giarrusso A., Eastmond G., Edwith A., Russo S., Sigwalt P. (1989). Conjugated Diene Polymerization. Comprehensive Polymer Science.

[2] Thiele S.K.H., Wilson D.R. (2003). Alternate Transition Metal Complex Based Diene Polymerization. J. Macromol. Sci. Polym. Rev..

[3] Friebe L., Nuyken O., Obrecht W. (2006). Neodymium-based Ziegler/Natta catalysts and their application in diene polymerization. Adv. Polym. Sci..

[4] Ricci G., Sommazzi A., Masi F., Ricci M., Boglia A., Leone G. (2010). Well Defined Transition Metal Complexes with Phosphorus and Nitrogen Ligands for 1,3-Dienes Polymerization. Coord. Chem. Rev..

[5] Ricci G., Pampaloni G., Sommazzi A., Masi F. (2021). Dienes Polymerization: Where we are and what lies ahead. Macromolecules.

[6] Huang J., Liu Z., Cui D., Liu X. (2018). Precisely Controlled Polymerization of Styrene and Conjugated Dienes by Group 3 Single-Site Catalysts. ChemCatChem.

[7] Srivastava V.K., Maiti M., Basak G.C., Jasra R.V. (2014). Role of catalysis in sustainable production of synthetic elastomers. J. Chem. Sci..

[8] Zhang Z., Cui D., Wang B., Liu B., Yang Y. (2010). Polymerization of 1,3-Conjugated Dienes with Rare-Earth Metal Precursors. Struct. Bond..

[9] Fischbach A., Anwander R. (2006). Rare-Earth Metals and Aluminum Getting Close in Ziegler-type Organometallics. Adv. Polym. Sci..

[10] Jothieswaran J., Fadlallah S., Bonnet F., Visseaux M. (2017). Recent Advances in Rare Earth Complexes Bearing Allyl Ligands and Their Reactivity towards Conjugated Dienes and Styrene Polymerization. Catalysts.

[11] Porri L., Giarrusso A., Ricci G. (1991). Recent views on the mechanism of diolefin polymerization with transition metal initiator systems. Prog. Polym. Sci..

[12] Liu B., Wang X., Pan Y., Lin F., Wu C., Qu J., Luo Y., Cui D. (2014). Unprecedented 3,4-isoprene and cis-1,4-butadiene copolymers with controlled sequence distribution by single Yttrium cationic species. Macromolecules.

[13] Tanaka R., Shinto Y., Nakayama Y., Shiono T. (2017). Synthesis of stereodiblock polybutadiene using Cp*Nd(BH_4_)_2_(thf)_2_ as a catalyst. Catalysts.

[14] Dong B., Liu H., Peng C., Zhao W., Zheng W., Zhang C., Bi J., Hu Y., Zhang X. (2018). Synthesis of stereoblock polybutadiene possessing cis-1,4 and syndiotactic-1,2 segments by imino-pyridine cobalt complex-based catalyst through one-pot polymerization process. Eur. Polym. J..

[15] Gong D., Ying W., Zhao J., Li W., Xu Y., Luo Y., Zhang X., Capacchione C., Grassi A. (2019). Controlling external diphenylcyclohexylphosphine feeding to achieve cis-1,4-syn-1,2 sequence controlled polybutadienes via cobalt catalyzed 1,3-butadiene polymerization. J. Catal..

[16] Bazzini C., Giarrusso A., Porri L. (2002). Diethylbis (2,2′-bipyridine) iron/MAO. A Very Active and Stereospecific Catalyst for 1,3-Diene Polymerization. Macromol. Rapid Commun..

[17] Ricci G., Morganti D., Sommazzi A., Santi R., Masi F. (2003). Polymerization of 1, 3-dienes with iron complexes based catalysts: Influence of the ligand on catalyst activity and stereospecificity. J. Mol. Cat. A Chem..

[18] Ricci G., Leone G., Boglia A., Bertini F., Boccia A.C., Zetta L. (2009). Synthesis and Characterization of Isotactic 1,2-Poly(E-3-methyl-1,3-pentadiene). Some Remarks about the Influence of Monomer Structure on Polymerization Stereoselectivity. Macromolecules.

[19] Ricci G., Battistella M., Porri L. (2001). Chemoselectivity and stereospecificity of chromium(II) catalysts for 1,3-diene polymerization. Macromolecules.

[20] Ricci G., Forni A., Boglia A., Sonzogni M. (2004). New chromium(II) bidentate phosphine complexes: Synthesis, characterization, and behavior in the polymerization of 1,3-butadiene. Organometallics.

[21] Bazzini C., Giarrusso A., Porri L., Pirozzi B., Napolitano R. (2004). Synthesis and characterization of syndiotactic 3,4-polyisoprene prepared with diethylbis(2,2’-bipyridine)iron−MAO. Polymer.

[22] Raynaud J., Wu J.Y., Ritter T. (2012). Iron-catalyzed polymerization of isoprene and other 1,3-dienes. Angew. Chem..

[23] Guo L., Jing X., Xiong S., Liu Y., Liu Z., Chen C. (2016). Influences of alkyl and aryl substituents on iminopyridine Fe(II)- and Co(II)-catalyzed isoprene polymerization. Polymers.

[24] Champouret Y., Hashmi O.H., Visseaux M. (2019). Discrete iron-based complexes: Applications in homogeneous coordination-insertion polymerization catalysis. Coord. Chem. Rev..

[25] Hashmi O.H., Champouret Y., Visseaux M. (2019). Highly active iminopyridyl iron-based catalysts for the polymerization of isoprene. Molecules.

[26] Zhao M., Wang L., Mahmood Q., Jing C., Zhu G., Zhang X., Wang X., Wang Q. (2019). Controlled isoprene polymerization mediated by iminopyridine-iron (II) acetylacetonate pre-catalysts. Appl. Organomet. Chem..

[27] Ricci G., Leone G., Zanchin G., Palucci B., Boccia A.C., Sommazzi A., Masi F., Zacchini S., Guelfi M., Pampaloni G. (2021). Highly Stereoregular 1,3-Butadiene and Isoprene Polymers through Monoalkyl−N−Aryl Substituted Iminopyridine Iron Complex-Based Catalysts: Synthesis and Characterization. Macromolecules.

[28] Eremina J.A., Lider E.V., Samsonenko D.G., Sheludyakova L.A., Berezin A.S., Klyushova L.S., Ostrovskii V.A., Trifonov R.E. (2019). Mixed-ligand copper(II) complexes with tetrazole derivatives and 2,2′- bipyridine, 1,10-phenanthroline: Synthesis, structure and cytotoxic activity. Inorg. Chim. Acta.

[29] Hernandez-Molina M., Gonzalez-Platas J., Ruiz-Perez C., Lloret F., Julve M. (1999). Crystal structure and magnetic properties of the single-m-chloro copper(II) chain [Cu(bipy)Cl2] (bipy = 2,2’-bipyridine). Inorg. Chim. Acta.

[30] Ricci G., Italia S., Giarrusso A., Porri L. (1993). Polymerization of 1,3-dienes with the soluble catalyst system methylaluminoxanes-[CpTiCl3]. Influence of monomer structure on polymerization stereospecificity. J. Organomet. Chem..

[31] Porri L., Ricci G., Giarrusso A., Shubin N., Lu Z., Arjunan P., McGrath J.C., Hanlon T. (2000). Recent developments in Lanthanide catalysts for 1,3-diene polymerization. ACS Symposium Series 749—Olefin Polymerization: Emerging Frontiers.

[32] Mochel V.D. (1972). Carbon-13 NMR of polybutadiene. J. Polym. Sci. Part A-1 Polym. Chem..

[33] Elgert K.F., Quack G., Stutzel B. (1974). Zur struktur des polybutadiens, 2. Das 13C-NMR-Spektrum des 1,2-polybutadiens. Makromol. Chem..

[34] Tanaka Y., Sato H. (1976). Sequence distribution of cis-1,4-and trans-1,4-units in polyisoprenes. Polymer.

[35] Sato H., Ono A., Tanaka Y. (1977). Distribution of isomeric structures in polyisoprenes. Polymer.

[36] Beebe D.H. (1978). Structure of 3,4-(cis-1,4-)trans-1,4-polyisoprene by 13C n.m.r. Polymer.

[37] Annunziata L., Pragliola S., Pappalardo D., Tedesco C., Pellecchia C. (2011). New (Anilidomethyl)pyridine Titanium(IV) and Zirconium(IV) Catalyst Precursors for the Highly Chemo- and Stereoselective cis-1,4-Polymerization of 1,3-Butadiene. Macromolecules.

[38] Pampaloni G., Guelfi M., Sommazzi A., Leone G., Masi F., Zacchini S., Ricci G. (2019). Synthesis and spectroscopic characterization of titanium pyridylanilido complexes as catalysts for the polymerization of 1,3-butadiene and isoprene. Inorg. Chim. Acta.

[39] Colamarco E., Milione S., Cuomo C., Grassi A. (2004). Homo- and copolymerization of butadiene catalyzed by a bis(imino)pyridyl vanadium compolex. Macromol. Rapid Commun..

[40] Leone G., Zanchin G., Pierro I., Sommazzi A., Forni A., Ricci G. (2017). Synthesis, structure and 1,3-butadiene polymerization behavior of vanadium (III) phosphine complexes. Catalysts.

[41] Ricci G., Forni A., Boglia A., Sommazzi A., Masi F. (2005). Synthesis, structure and butadiene polymerization behavior of CoCl_2_(PR_x_Ph_3-x_)_2_ (R = methyl, ethyl, propyl, allyl, isopropyl, cyclohexyl; x = 1,2). Influence of the phosphorous ligand on polymerization stereoselectivity. J. Organomet. Chem..

[42] Ricci G., Sommazzi A., Leone G., Boglia A., Masi F. (2013). Bis- imine complex of lanthanide, catalytic system comprising said bis- imine complex and process for the (co)polymerization of conjugated dienes.

