# Supplementary material for: Dichloro(2,2′-bipyridine)copper/MAO: An Active and Stereospecific Catalyst for 1,3-Diene Polymerization
Source: Molecules. 2023 Jan 2;28(1):374. doi: 10.3390/molecules28010374 (PMC9822443; doi:10.3390/molecules28010374)

SUPPORTING INFORMATION

Dichloro(2,2′-bipyridine)copper/MAO. An Active and Stereospecific Catalyst for 1,3-Diene Polymerization

Giovanni Ricci,[a,*] Giuseppe Leone,[a] Giorgia Zanchin,[a] Francesco Masi,[b] Massimo Guelfi,[c] Guido Pampaloni[c]

[a] CNR-Istituto di Scienze e Tecnologie Chimiche "Giulio Natta" (SCITEC), via A. Corti 12, I-20133 Milano, Italy.
[b] Scientific Advisor, Via Galvani 7, I- 26866 Sant'Angelo Lodigiano (LO), Italy.
[c] Università di Pisa, Dipartimento di Chimica e Chimica Industriale, Via Moruzzi 13, I-56124 Pisa, Italy.
* Correspondence: giovanni.ricci@scitec.cnr.it

Table of Contents

Figure S1. IR spectrum (solid state) of Cu(bipy)Cl_2 prepared in toluene.

Figure S2. IR spectrum (solid state) of Cu(bipy)Cl_2 prepared in ethanol.

Figure S3. Superposition of IR spectra (solid state) of Cu(bipy)Cl_2 prepared in ethanol/toluene.

Figure S4 – S13. FTIR spectra of the obtained polymers.

Figure S14 – S22. ^{1}H and ^{13}C NMR spectra of the obtained polymers.

Figure S1. IR spectrum (solid state) of **Cu(bipy)Cl₂** prepared in toluene.

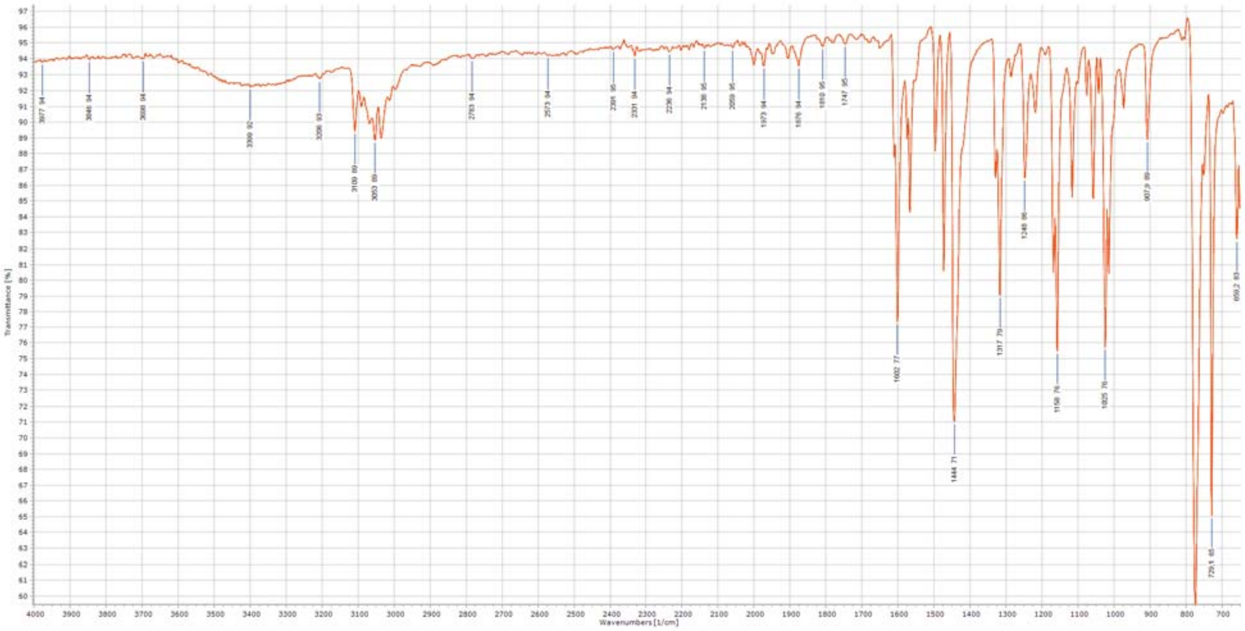

Figure S2. IR spectrum (solid state) of **Cu(bipy)Cl₂** prepared in ethanol.

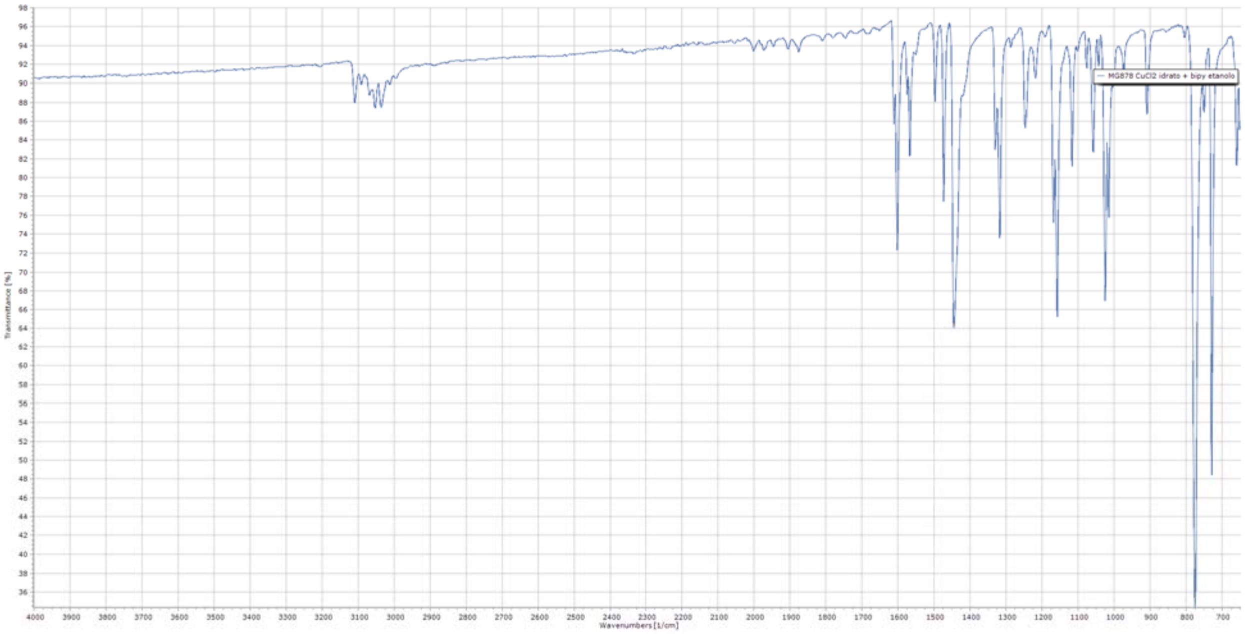

Figure S3. Superposition of IR spectra (solid state) of **Cu(bipy)Cl₂** prepared in ethanol/toluene.

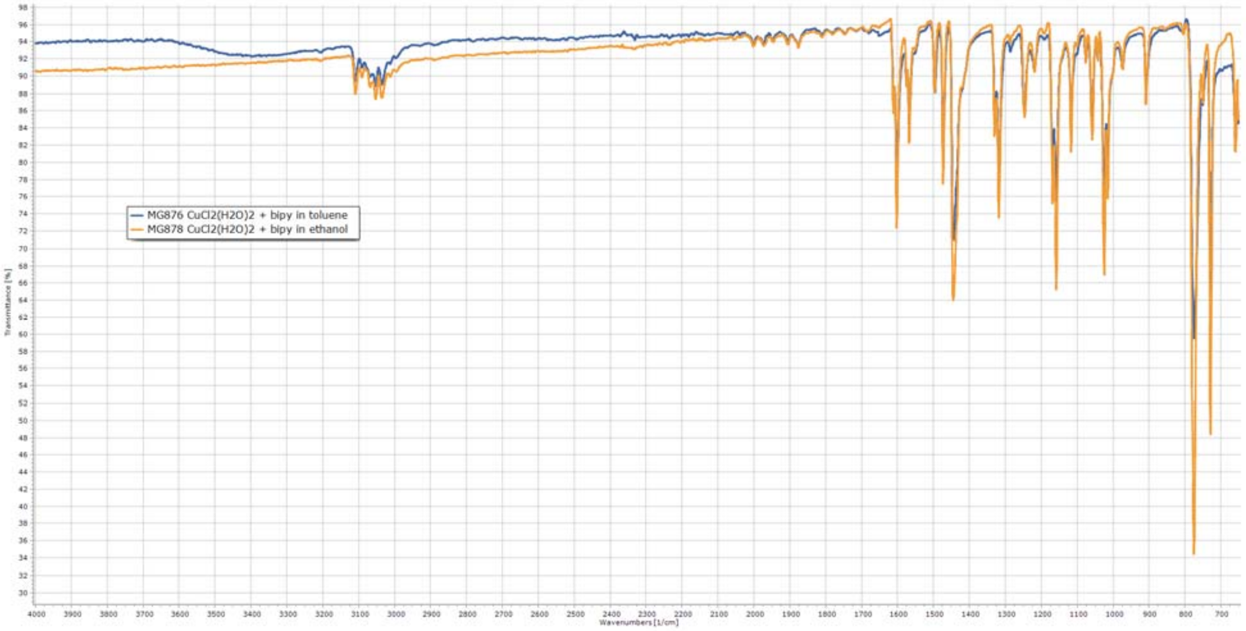

Figure S4. FTIR spectrum of entry **1** (ref. Table 1 of the manuscript).

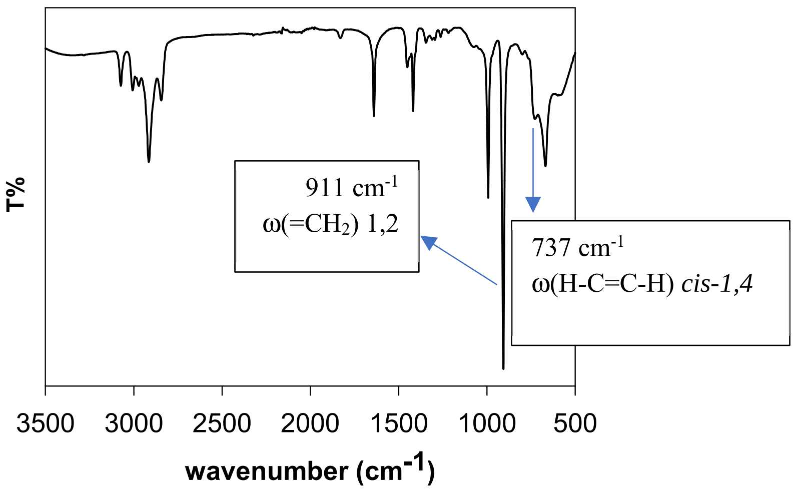

Figure S5. FTIR spectrum of entry **2** (ref. Table 1 of the manuscript).

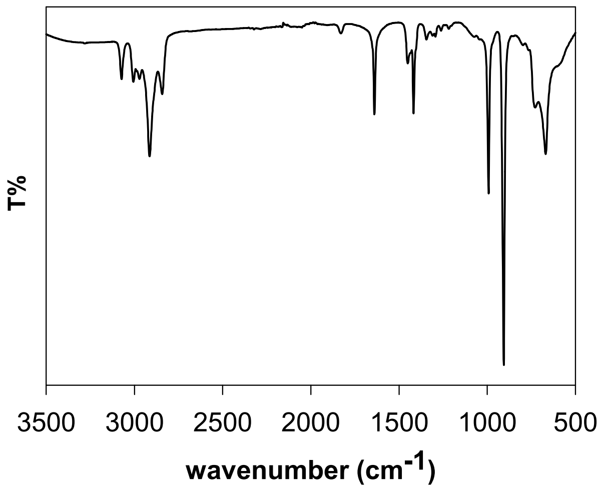

Figure S6. FTIR spectrum of entry **3** (ref. Table 1 of the manuscript).

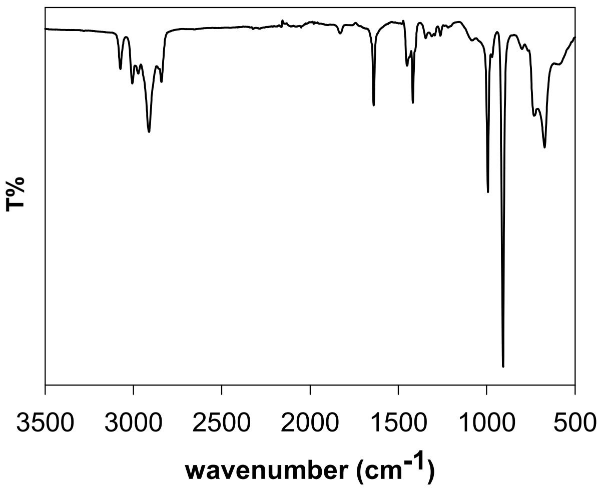

Figure S7. FTIR spectrum of entry **4** (ref. Table 1 of the manuscript).

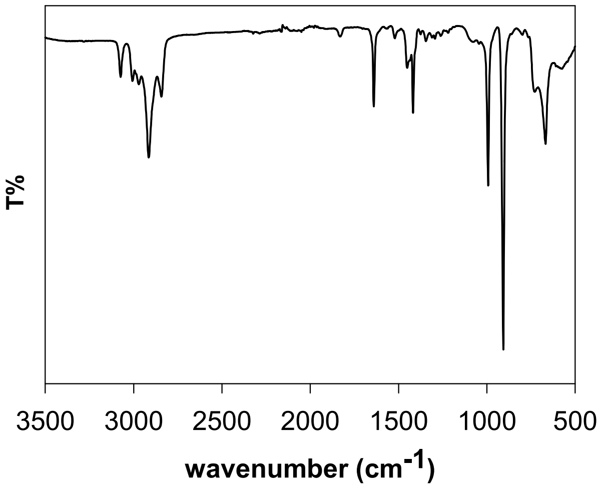

Figure S8. FTIR spectrum of entry **5** (ref. Table 1 of the manuscript).

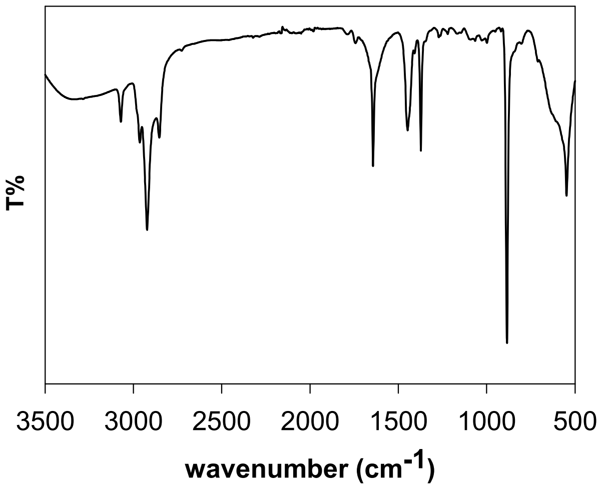

Figure S9. FTIR spectrum of entry **6** (ref. Table 1 of the manuscript).

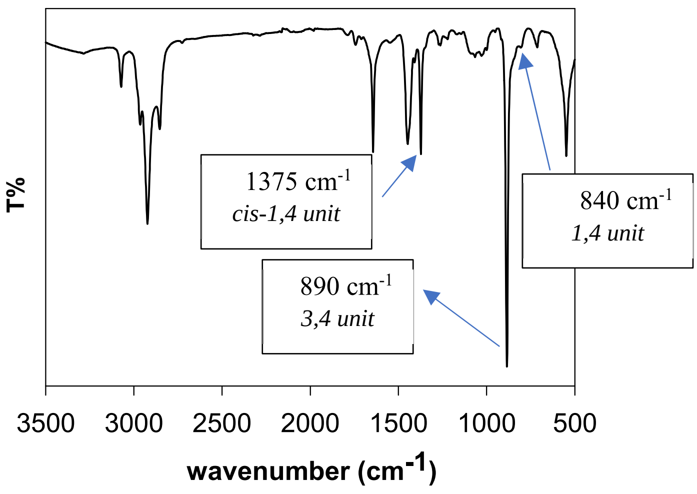

Figure S10. FTIR spectrum of entry **7** (ref. Table 1 of the manuscript).

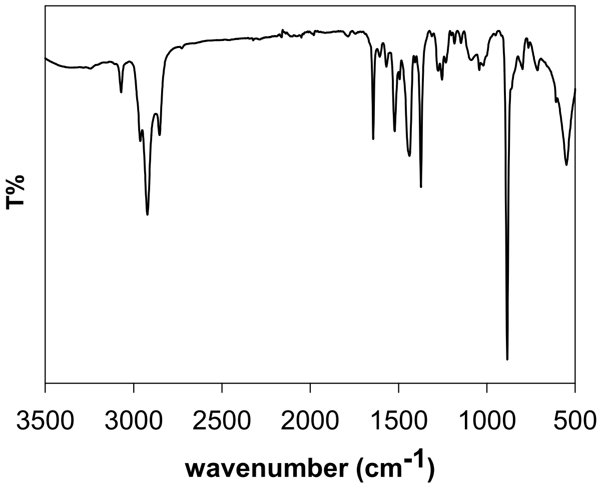

Figure S11. FTIR spectrum of entry **8** (ref. Table 1 of the manuscript).

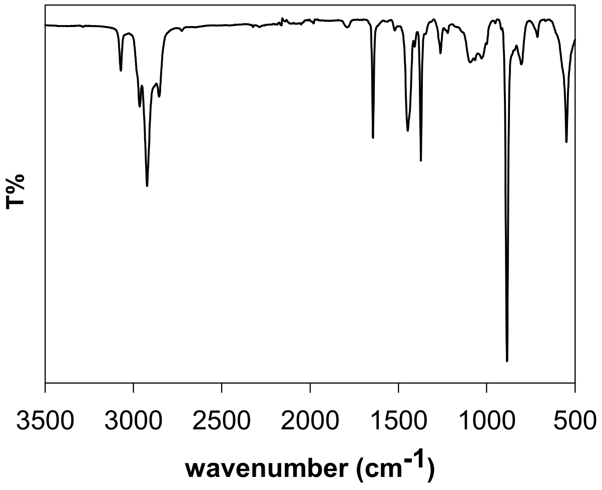

Figure S12. FTIR spectrum of entry **9** (ref. Table 1 of the manuscript).

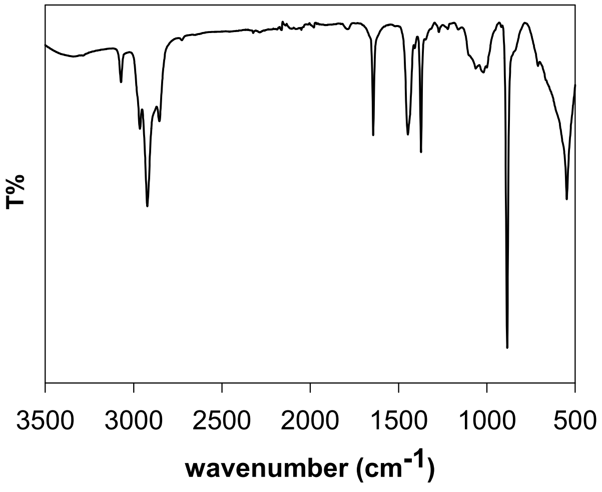

Figure S13. FTIR spectrum of entry **10** (ref Table 1 of the manuscript).

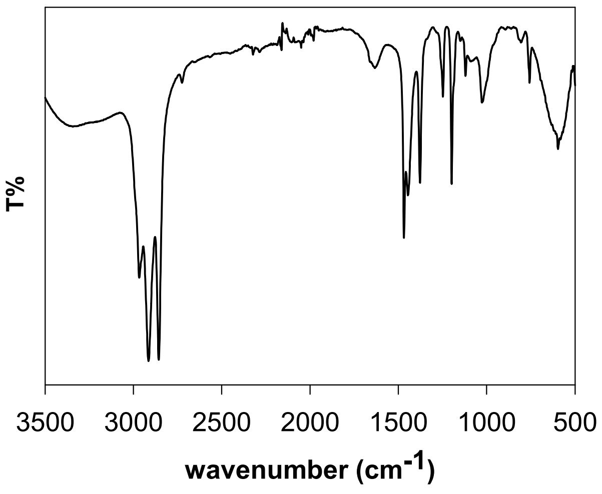

Figure S14. ^{1}H (bottom) and ^{13}C NMR (top) spectra of entry **1** (ref. Table 1 of the manuscript).

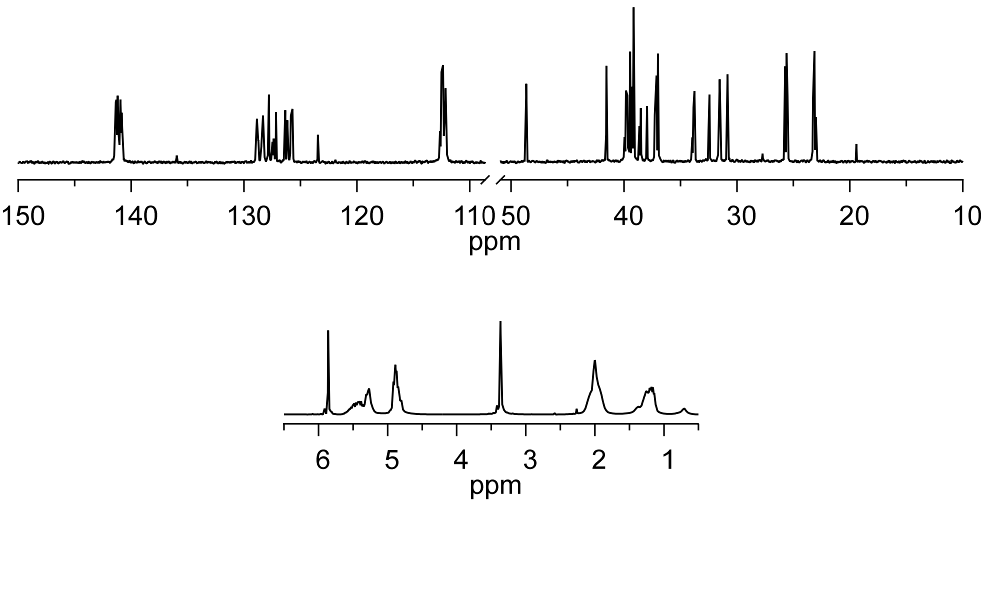

Figure S15. ^{1}H (bottom) and ^{13}C NMR (top) spectra of entry **2** (ref. Table 1 of the manuscript).

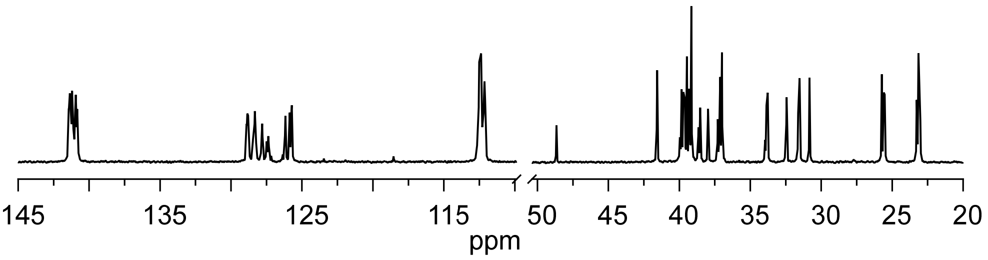

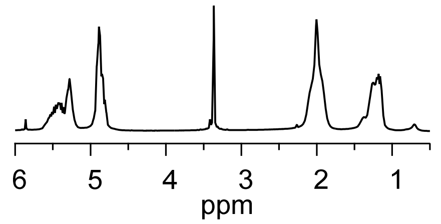

Figure S16. ^{1}H (bottom) and ^{13}C NMR (top) spectra of entry **3** (ref. Table 1 of the manuscript).

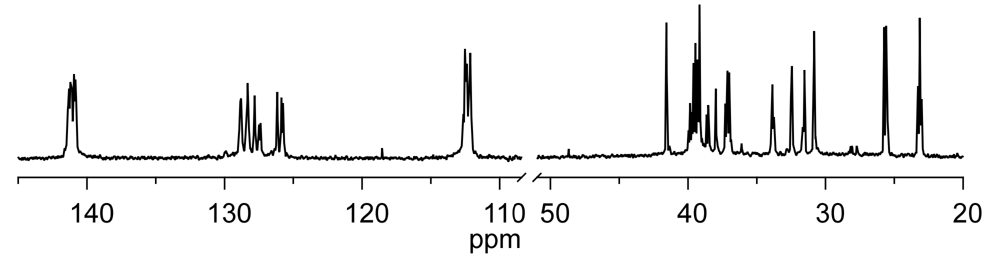

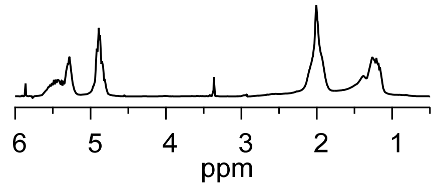

Figure S17. ^{1}H (bottom) and ^{13}C NMR (top) spectra of entry **4** (ref. Table 1 of the manuscript).

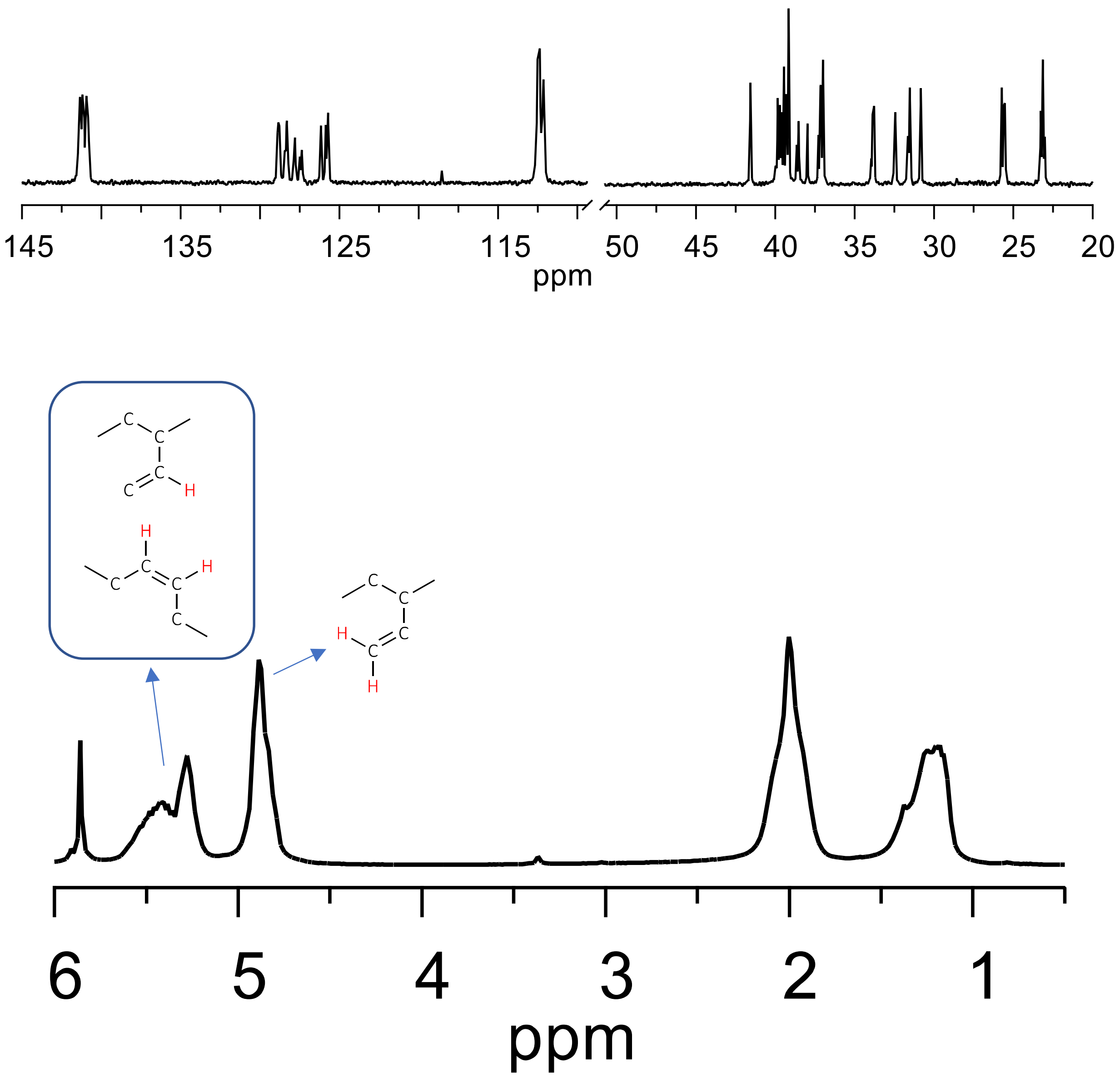

Figure S18. ^{1}H (bottom) and ^{13}C NMR (top) spectra of entry **5** (ref. Table 1 of the manuscript).

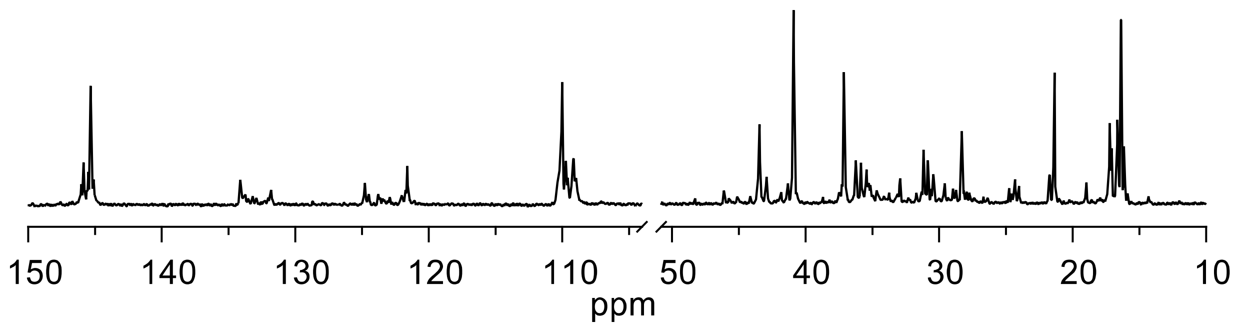

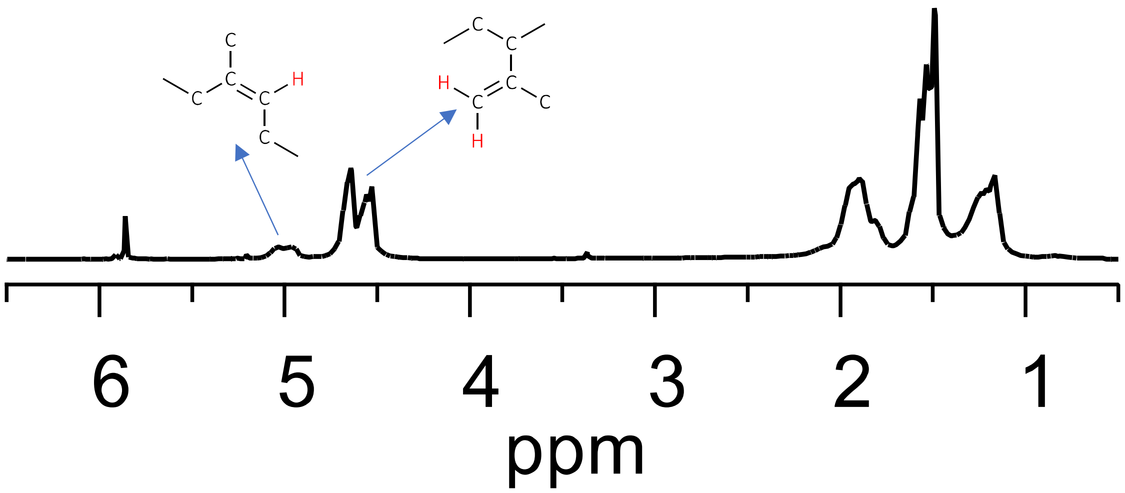

Figure S19. ^{1}H (bottom) and ^{13}C NMR (top) spectra of entry **6** (ref. Table 1 of the manuscript).

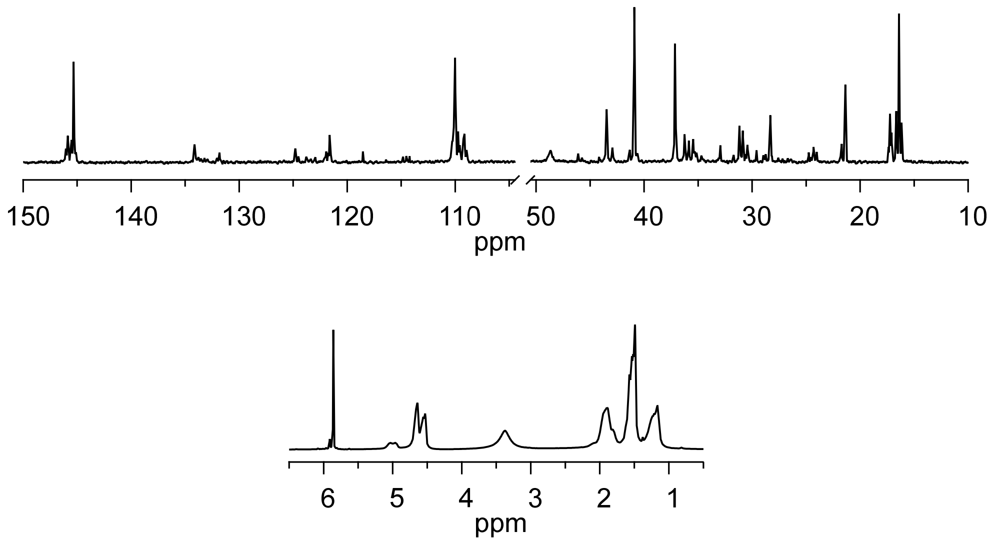

Figure S20. ^{1}H (bottom) and ^{13}C NMR (top) spectra of entry **7** (ref. Table 1 of the manuscript).

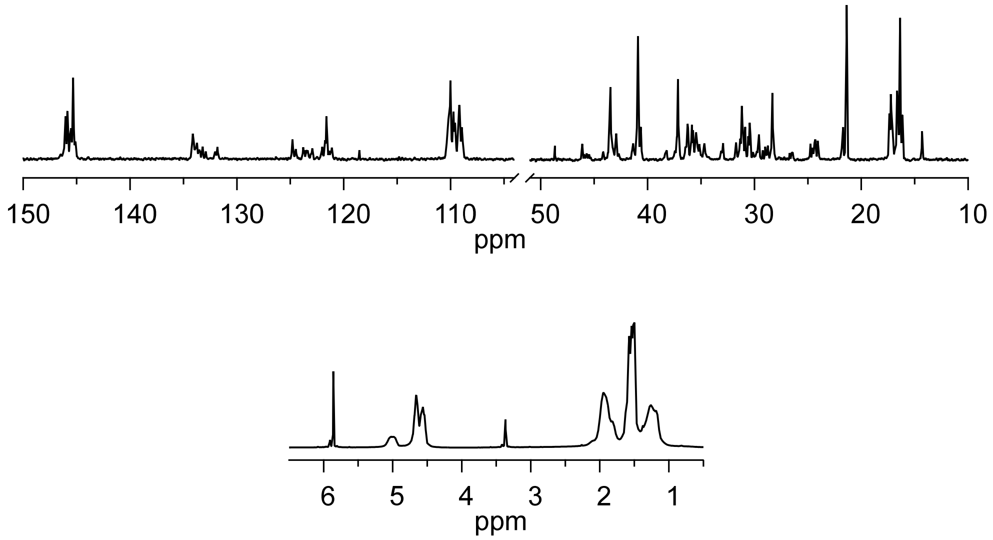

Figure S21. ^{1}H (bottom) and ^{13}C NMR (top) spectra of entry **8** (ref. Table 1 of the manuscript).

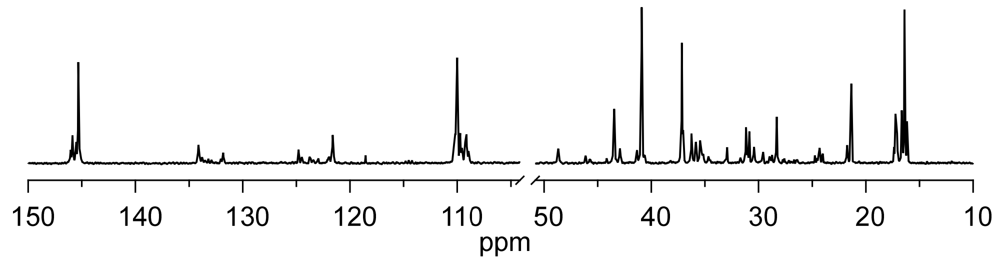

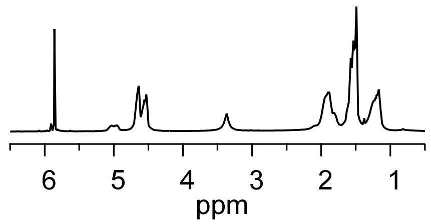

Figure S22. ^{1}H (bottom) and ^{13}C NMR (top) spectra of entry **9** (ref. Table 1 of the manuscript).

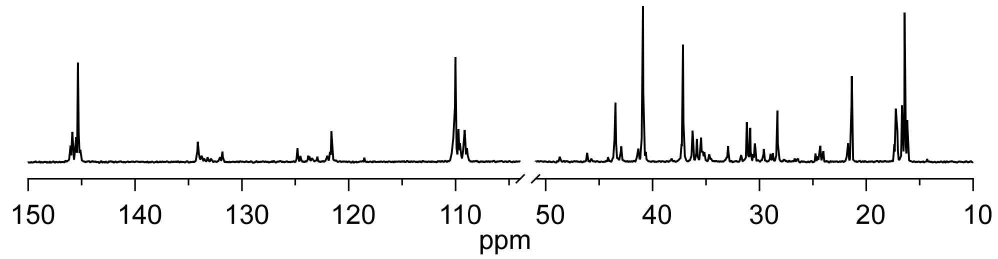

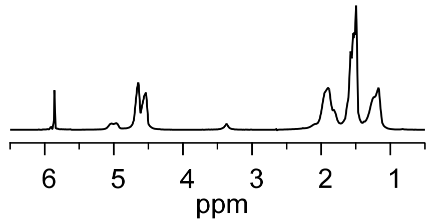

Supplement: Supplementary file 1 [file molecules-28-00374-s001.zip › molecules-2106095-supplementary.pdf]
